# The competence of nurses caring for COVID-19 patients regarding disaster management: Structural equation modeling of knowledge, attitude, and performance

**DOI:** 10.1016/j.heliyon.2024.e35568

**Published:** 2024-08-02

**Authors:** Maryam Khandan, Zinab Ghorbani, Mohsen Golestani, Faranak Moradi

**Affiliations:** a Department of Nursing, Faculty of Nursing and Midwifery, Kerman Branch, Islamic Azad University, Kerman, Iran; b Department of Nursing and Midwifery, Kerman Branch, Islamic Azad University, Kerman, Iran

**Keywords:** Competence, Knowledge, Attitude, Performance, Nurses, COVID-19, Disaster management

## Abstract

**Background:**

The outbreak of COVID-19 as a global disaster has required nurses, as front-line providers of safe and quality care to patients with this disease, for proper disaster management to have a high level of competence, which demands an acceptable level of knowledge, attitude, and performance. Therefore, this study was conducted to model the relationship between knowledge, attitude, and performance with the competence of nurses caring for patients with COVID-19 regarding disaster management.

**Methods:**

This descriptive-correlational study was conducted on 291 hospital nurses using the structural equation modeling approach in three provinces of Iran, including Kerman, Hormozgan, and Fars, from June to December 2021. Data were collected through a researcher-made questionnaire that provided information on demographics, knowledge, attitude, performance, and competence regarding disaster management. Data analysis, which included descriptive and inferential statistics, was performed using SPSS and AMOS software. Moreover, the structural equation modeling method was based on the covariance to examine the proposed model of the relationship between knowledge, attitude, and performance with nurses’ competence.

**Results:**

The study revealed that the nurses had average knowledge, acceptable attitudes, high performance, and acceptable competence scores. A positive and significant relationship was found between knowledge, attitude, and performance with nurses' competencies (P < 00.05). Furthermore, the coefficient of determination of nurses' competence in the modified structural model indicates that exogenous variables, namely knowledge, attitude, and performance, could predict 36 % of the changes in nurses' competence. Finally, the path coefficient of the effect of knowledge, attitude, and performance on nurses’ competence was higher than 1.96.

**Conclusions:**

According to the study, although the knowledge, attitude, performance, and competence of nurses caring for patients with COVID-19 were at an acceptable level, there were obstacles to improving their competence. Hence, identifying and prioritizing educational needs and learning preferences based on their cultural backgrounds are also emphasized.

## 1 Introduction

One of the critical conditions the world is dealing with is the COVID-19 outbreak, which has been described as the most demanding health disaster and the most significant challenge since World War II [1]. As of April 19, 2021, the global COVID-19 infection count was over 142 million, with more than 3 million fatalities. Similar to many other countries, Iran has been severely impacted by the pandemic, and statistics indicate that it is among the top ten nations with the highest number of cases and deaths. As of September 9, 2023, the total number of confirmed COVID-19 cases in Iran stands at 7,613,757, with 146,329 deaths [2]. The International Council of Nurses (ICN) has emphasized nurses’ crucial role during emergencies and disasters. The COVID-19 pandemic has presented several challenges for them due to the unprecedented pressure on healthcare systems. These challenges include a surge in the number of patients, an increased burden of disease, and the need to follow protective protocols [3].

Competency refers to a person's actual performance in a specific role and situation [4]. Moreover, this concept refers to a combination of knowledge, skills, abilities, and behavior necessary for performing a specific job or task [5]. Despite many efforts to prepare healthcare providers for disasters, studies indicate limited effectiveness [6]. Hence, ensuring professional competency is crucial for providing quality healthcare services. The rapid spread of this disease involved a tremendous human force in its prevention and treatment [7] and required nurses to be on the frontline of direct care for infected patients [1]. The results of several studies indicate that nurses' preparedness, knowledge, attitude, and performance in the face of crises are at a low level, causing improper management of crises and disasters [8,9]. In other studies, nurses' level of preparedness in crises and disasters has been reported as moderate to weak [10,11]. Similar results indicate nurses' insufficient preparedness and confidence, suboptimal competence, and unacceptable knowledge and performance levels to respond to disasters [[12], [13], [14], [15]].

On the other hand, nurses are required to take several roles and performances, including health training, screening services, supporting the general public and a high-risk group of people, preventing and monitoring hospital infections, taking appropriate measures and preparations for the older people, supporting patients with immunodeficiency and chronic diseases, and providing care for patients with COVID-19 [16,17]. Therefore, providing high-quality and safe care as a part of the patient's rights requires a fully committed team. In addition, it is a moral imperative to ensure a competent and compassionate workforce that continuously provides highly reliable health care [18]. Iranian nurses have been performing their care duties during disasters in high-risk situations [19]. It is crucial to ensure that healthcare workers are adequately prepared to respond to disasters to safeguard themselves and the public. According to a recent study, not only civilians but also numerous healthcare workers worldwide have lost their lives due to COVID-19 [20]. One possible reason for nurses lacking emergency and disaster-preparedness competencies is their inadequate knowledge and experience in disaster-planning skills, implementing disaster guidelines, and assessing patients in disaster situations [21]. Additionally, nurses' competence is greatly affected by various factors, including their proficiency in disaster management and ability to serve on the front lines of the battle against emerging infectious diseases such as COVID-19. Identifying these factors can help develop effective educational programs and policies to enhance the professional competencies of nurses and manage the workforce efficiently [22]. To acquire core competencies in disaster, nurses need knowledge, skills, and attitudes toward disaster preparedness. Previous studies reveal a significant correlation between nurses' disaster core competencies and their knowledge, skills, and experience [12,23].

Hence, it remains unclear whether nurses have sufficient competence to manage this emerging phenomenon, whether their knowledge, attitude, and performance are adequate to provide health services, and how these concepts affect their competence. Therefore, the study aims to measure the three independent variables of knowledge, attitude, and performance. Each of which measures an independent objective. Moreover, the literature review showed that these independent variables could impact the dependent variable (competence) in some ways. Therefore, it was important to determine how much and how each independent variable could predict the dependent ones. Hence, firstly, by measuring the nurses' knowledge, attitude, and performance as independent variables and their competence as a dependent variable, then considering the importance of nurses’ role in managing the COVID-19 disaster, this study was conducted to model the relationship between knowledge, attitude, and performance with the competence of nurses caring for patients with COVID-19 regarding disaster management in Kerman, Hormozgan, and Fars, Iran.

## 2 Materials and methods

### 2.1 Study design and setting

This descriptive-correlational study was carried out with the structural equation modeling approach in hospitals affiliated with the University of Medical Sciences in the capital of Kerman, Hormozgan, and Fars, Iran, from June to December 2021. The hospitals included Afzalipour, Shahid Mohammadi, and Ali Asghar, selected as the major referral centers for patients with COVID-19 receiving treatment and follow-up.

### 2.2 Participants and procedure

The list of nurses who were directly involved in the care of patients hospitalized in the COVID-19 wards of these three hospitals (N = 821) was obtained from nursing managers. The inclusion criteria included the willingness to participate and at least six months of work experience in the ward providing health services to patients with COVID-19. Participants were recruited using the purposive sampling method. The sample size was calculated as 280 participants using G*Power software (Effect size f = 0.3, α = 0.05). Considering 20 % sample attrition, informed consent forms and questionnaires were sent to at least 340 individuals via a link.

Permission to conduct the study was obtained from the Research Ethics Committee of Kerman University of Medical Sciences. Afterward, in order to collect the data, the researchers attended the ward caring for patients with COVID-19 in hospitals affiliated with medical sciences in the capital of three provinces, including Kerman, Hormozgan, and Fars. After introducing themselves, the researchers explained the purpose of the study to the relevant authorities. Afterward, if the nurses were willing to participate in the study, there was a possibility to fill out the written informed consent form and the questionnaires manually. However, if desired, a link to complete all electronic records and questionnaires was provided to the participants.

### 2.3 Data collection tools and variable measurement

Data were collected using a four-part survey of demographic information and a researcher-made questionnaire of knowledge, attitude, performance, and competence based on a literature review [9,12,14,[24], [25], [26], [27], [28], [29]]. First, the initial draft of items was created. In order to determine the quantitative face validity using the “item impact” method, the opinions of ten nurses working in the COVID-19 wards were used. The items with an impact score higher than 1.5 were recognized as suitable for further analysis and retained. In the next step, the content validity was quantitatively evaluated using the Content Validity Ratio (CVR) and the Content Validity Index (CVI), and the items were sent to ten nursing and midwifery professors via emails, only 8 of whom responded. The other results are presented in Table 1. The final tool was designed in five sections. The first section included nurses’ demographic information and a few multiple-choice questions related to issues such as the responsibility of caring for patients in disaster, participation in courses or workshops on disaster management, the need for training on disaster management, preferences, and educational needs.

**Table 1:** Validity indicators and the number of questionnaire items.

Variable	CVR	CVI	The number of primary items	The number of final items	S-CVI/UA	S-CVI/Ave
Knowledge	0.95	0.89	35	31	0.88	**0.83**
Attitude	1	1	18	18	1	**1**
Performance	0.98	0.95	20	19	0.93	**0.88**
Competence	0.97	0.92	25	23	0.89	**0.86**

The second section included 31 items to assess nurses' knowledge of disaster management in seven parts, including “disaster management system and the nurse's role in it,” “triage,” “documentation and correspondence,” “psychological issues and specific populations,” “isolation,” “sanitizing and quarantine,” “reporting and access,” and “the biological factors.” Responses were scored from 4 = Very high to 1 = Very low, with a maximum score of 124, a minimum score of 31, and a mean score of 31–60 = low, 61–92 = average, and 93–124 = high knowledge level. The third section, which included 18 options, investigated the attitude toward disaster management. The responses ranged from ‘completely agree’ to ‘completely disagree’ (5–1), with maximum and minimum scores of 90 and 18, respectively. A score of 18–41 was considered weak, 42–65 = average, and 66–90 = acceptable levels of attitude. The fourth section assessed the performance concerning disaster management. The responses were selected from among ‘always,’ ‘often,’ ‘sometimes,’ ‘rarely,’ and ‘never’ (score 5 to 1), with a maximum and a minimum score of 95 and 19, respectively. The mean score of 19–43 was considered weak, 44–69 = medium, and 70–95 = high levels of performance. The fifth section measured nurses' competence regarding disaster management and included 23 items. The responses ranged from ‘very important’ to ‘very unimportant’ (5–1), with the maximum and the minimum scores of 115 and 23, respectively. The mean score between 23 and 53 was considered weak, 54–84 = average, and 85–115 = acceptable levels of competencies. In order to determine the reliability, Cronbach's alpha coefficient of knowledge, attitude, performance, and competence questionnaires was calculated as 0.98, 0.99, 0.88, and 0.97, respectively.

### 2.4 Data analysis

The collected data was analyzed using SPSS_V20_ software. Demographic data was described using mean (SD) and frequency (percentage). Independent samples *t*-test, analysis of variance, and Pearson's correlation coefficient were used to compare demographic variables with nurses' knowledge, attitude, performance, and competence. Due to the skewness and kurtosis of the variables in the range of ±2 and ± 3, the univariate normality was confirmed. Moreover, multivariate normality was obtained at −0.237 and −0.113 using Mardia standardized kurtosis coefficient and critical ratio, respectively (<5). Based on the d2 index and significance levels less than 0.05, 74 data were identified as outliers. Before evaluating the structural models, it is crucial to examine their presuppositions. One of the prerequisites is to analyze the multivariate outlier data using Mahalanobis distance. Based on this index, 74 samples were identified during the structural model implementation and excluded. However, in the descriptive part, all participants' information is reported. The non-multicollinearity assumption was also evaluated using the tolerance indices and variance inflation factor (VIF).

Moreover, no deviation from the multicollinearity assumption was observed in the calculated values of the tolerance statistics and variance inflation factor. Therefore, in order to test the proposed model of the relationship between knowledge, attitude, and performance with nurses’ competence, the structural equation modeling method based on the covariance method was used employing AMOS software version 24, and model parameters were estimated by the Maximum Likelihood (ML) method. In order to assess the characteristics of the fit of the model, chi-square, the Degree of Freedom Ratio (CMIN/DF), Parsimonious Normal Fit Index (PNFI), Comparative Fit Index (CFI), Parsimonious Comparative Fit Index (PCFI), Incremental Fit Index (IFI), Goodness of Fit Index (GFI), and Root Mean Square Error of Approximation (RMSEA) were used [30]. The fit indices presented in Table 5 reveal an acceptable fit for the modified model.

Data analysis revealed that out of all the demographic variables, only two variables, namely Position and Work experience, had a relationship with competence. However, when they were included in the model, goodness-of-fit indices became too weak. Moreover, they did not have a significant relationship with the Competence variable in the model. Hence, to control the effect of these variables on competence, they were not included in the model.

## 3 Results

Of the 340 invitations sent through the link, 291 nurses completed the informed consent form and the questionnaires. This study's findings showed that nurses' knowledge, attitude, performance, and competence scores were at average, acceptable, high, and acceptable levels, respectively (Table 2).

**Table 2:** Descriptive indicators of nurses’ knowledge, attitude, performance, and competence.

Variable	Mean (SD)	Maximum-Minimum	Skewness	Kurtosis	The total score		
					Poor N (%)	Average N (%)	Acceptable N (%)
Knowledge	90.12(18.70)	124–31	−0.242	−0.156	12(4.1)	154(52.9)	125(43)
Disaster management system and the nurse's role in it	13.6(3.11)	20–5	−0.028	0.088			
Triage	12.03(2.60)	16–4	−0.240	−0.369			
Documentation and correspondence	13.75(3.39)	20–5	−0.241	−0.018			
Psychological issues and special demographics	14.02(2.91)	20–5	−0.234	−0.375			
Isolation, sanitizing, and quarantine	12.10(2.91)	16–4	−0.348	−0.646			
Reporting and access	15.21(3.68)	20–5	−0.393	−0.463			
Biological factors	9.36(2.27)	12–3	−0.571	−0.165			
Attitude	73.37(9.26)	90–43	−0.634	−0.056	–	52(17.9)	239(82.1)
Performance	70.44(13.41)	95–34	−0.182	−0.283	8(2.7)	130(44.7)	153(52.6)
Competence	101.22(11.22)	115–46	1.05	2.88	2(0.7)	16(5.5)	273(93.8)

*Note: Categorizing the levels is based on the cutting point of the research tool.

The participants’ demographic characteristics and their comparison with the variables of knowledge, attitude, performance, and competence are presented in Table 3.

**Table 3:** The participants’ demographic characteristics and comparison of knowledge, attitude, performance and competence level (N = 291).

Variable		N (%)	Knowledge	Attitude	Performance	Competence
			Mean ± SD			
Gender	Female	218(74.9)	87.51 ± 17.66	74 ± 9.51	69.58 ± 13.12	100.83 ± 13.60
	Male	73(25.1)	97.91 ± 19.67	71.52 ± 8.27	73 ± 14.02	100.06 ± 10.31
*t(p)*			−4.22(0.001)	1.98(0.040)	−1.98(0.06)	0.44(0.66)
Marital status	Single	107(36.8)	91.56 ± 18.59	72.41 ± 9.22	70.80 ± 12.91	99.78 ± 12.79
	Married	184(63.2)	89.29 ± 18.77	73.94 ± 9.26	70.23 ± 13.72	101.14 ± 12.88
*t(p)*			0.99(0.32)	−1.35(0.17)	0.34(0.72)	−0.86(0.38)
Age (Year)	18–27	64(22)	86.75 ± 16.13	71.70 ± 10.01	69.06 ± 11.72	97.90 ± 11.72
	28–37	134(46)	88.41 ± 17.30	72.79 ± 9.44	68.20 ± 13.20	100.49 ± 12.23
	>37	93(32)	94.91 ± 21.36	75.36 ± 8.15	74.61 ± 13.95	102.74 ± 14.13
*F*(*p*)			4.76(0.009)	3.50(0.03)	6.96(0.001)	2.73(0.06)
Education	MSc^a^	266(91.4)	89.75 ± 17.41	73.28 ± 9.17	69.56 ± 12.84	100.52 ± 11.94
	BSc^b^	25(8.6)	94.04 ± 29.32	74.36 ± 10.29	79.86 ± 15.88	101.92 ± 20.42
*t(p)*			−1.09(0.27)	−0.55(0.58)	−3.7(<0.001)	−0.51(0.60)
Position	Staff nurse	245(84.2)	88.60 ± 17.64	72.48 ± 9.13	68.72 ± 12.71	99.64 ± 12.80
	Head nurse	29(10)	95.62 ± 233.64	77.82 ± 8.25	77.93 ± 14.11	105.03 ± 12.13
	Supervisor	17(5.8)	102.76 ± 18.86	78.80 ± 9.23	82.47 ± 12.03	107.47 ± 111.07
*F(p)*			6.15(0.002)	7.63(<0.001)	14.6(<0.001)	4.96(0.008)
Workplace	Emergency wards	113(38.8)	93.46 ± 15.2	73.53 ± 9.27	70.26 ± 12.79	100.81 ± 10.74
	Internal wards	91(31.3)	88.37 ± 21.93	73.67 ± 9.33	70.29 ± 13.56	100.64 ± 11.74
	ICU^c^	87(29.9)	87.62 ± 18.67	72.86 ± 9.27	70.82 ± 14.17	100.41 ± 16.17
*F(p)*			3.02(0.05)	0.19(0.82)	0.05(0.95)	0.02(0.97)
Work Experience (years) Mean ± SD	9.86 ± 6.80		*r* = 0.125 *p* = 0.03	*r* = 0.199 *p* = 0.001	*r* = 0.197 *p* = 0.001	*r* = 0.157 *p* = 0.007

a: Master of Science; b: Bachelor of Science; c:Intencive Care Unit.

*t*: Independent t; *F*: Analysis of variance, *r*: Pearson correlation coefficient.

The results indicated a statistically significant difference between nurses’ age and knowledge, attitude and performance, education level and performance, gender and knowledge and attitude, and between position and knowledge, attitude, and performance (P < 0.05). Moreover, there was a positive and significant relationship between work experience and knowledge, attitude, and competence (P < 0.05). Background information is provided in Table 4.

**Table 4:** Frequency distribution of background characteristics of participants (N = 291).

Background characteristics	Answer	N (%)
History of responsibility for caring for patients in critical situations	Yes	256(88)
	No	35(12)
Facing critical conditions in the last 10 years	Never	29(10)
	once	92(31.6)
	2-5 times	90(30.9)
	More than 5 times	80(27.5)
Participation in disaster management exercises in the last 10 years	Never	69(23.7)
	once	100(34.4)
	2-5 times	92(31.6)
	More than 5 times	30(10.3)
Participation in disaster management training courses	Yes	251(86.3)
	No	40(13.7)
Feeling a need to receive training in disaster management	Yes	254(97.3)
	No	37(12.7)
Having an official certificate of disaster management	Yes	82(28,2)
	No	209(71.8)
Participation in courses and workshops related to disaster management in the last 10 years	Never	37(12.7)
	once	93(32)
	2-5 times	108(37.1)
	More than 5 times	53(18.2)
Responsible for providing necessary training on disaster management	Nursing schools	19(6.5)
	Hospital Education Department	68(23.4)
	Both of the above	204(70.1)
Passing the academic credit related to disaster management	Yes	154(52.9)
	No	137(47.1)
Receiving adequate training in disaster management	Yes	105(36.1)
	No	186(63.9)

**Table 5:** The fit indices of the proposed and modified model in the present study.

Fit indices of the model	$x^{2}$	df	P-Value	CMIN/Df	RMSEA	PNFI	CFI	PCFI	IFI	GFI
Proposed model	102.016	32	<0.001	3.188	0.1	0.647	0.924	0.657	0.924	0.873
Modified model	62.586	27	<0.001	2.318	0.078	0.581	0.982	0.589	0.982	0.948

CMIN/DF: Chi-square/degree-of-freedom ratio; RMSEA: Root Mean Square Error of Approximation; PCFI: Parsimonious Comparative Fit Index; GFI: Goodness of Fit Index; PNFI: Parsimonious Normed Fit Index; IFI: Incremental Fit Index; CFI: Comparative Fit Index. Acceptable level of indices: PNFI, PCFI, (>0.5); CFI, GFI, IFI (>0.9), RMSEA (<0.08), CMIN/DF (good <3, acceptable >5).

The results of Spearman's correlation matrix showed a positive and significant relationship between knowledge (r = 0.260), attitude (r = 0.475), and performance (r = 0.439) with nurses' competencies (P < 0.05). Finally, prior to assessing the structural coefficients, the fit of the proposed model was evaluated based on the introduced goodness-of-fit indices. Considering that the CMIN/DF values were less than five and the RMSEA was less than 0.1, the fit of the proposed model was confirmed. In order to improve the model, it was modified by releasing several covariance errors. After the modifications, the results showed that the final model had a good fit, and the indices of PNFI, PCFI (>0.5, CFI, GFI, IFI (>0.9), RMSEA (<0.08), CMIN/DF (3 > good, 5 > acceptable) were acceptable. The R^2^ index shows the explained variance of endogenous latent variables. The values of R^2^, 0.26, 0.13, and 0.02 in structural equations as strong, medium, and weak, respectively (Table 5).

Furthermore, the coefficient of determination of nurses' competence in the modified structural model was 0.360, indicating that exogenous variables, namely knowledge, attitude, and performance, could predict 36 % of the changes in nurses' competence. Therefore, regarding the estimated indices, the structural relationship between knowledge, attitude, and performance with the nurses' competence had a good fit. Table 6 shows the results of the direct relationships between research variables in the final model. All path coefficients were statistically significant in the whole sample, and the relationship between all variables was positive and significant. The results show that the path coefficient of the effect of knowledge, attitude, and performance on nurses' competence is higher than 1.96. Consequently, it can be stated that knowledge, attitude, and performance affect the nurses’ competence significantly and positively. In addition, the direct effect of Knowledge, Attitude, and Performance variables on Competence was equal to 0.205, 0.380, and 0.330, respectively. Consequently, by increasing one unit of Knowledge, Attitude, and Performance variables, the level of Competence increased by 0.205, 0.380, and 0.330, respectively.

**Table 6:** The standard coefficients of the paths of the final model.

Path	Standardized Coefficients	Standard Error	Critical Ratio	*P*
Knowledge →Competence	0.205	0.045	2.337	0.012
Attitude→ Competence	0.380	0.070	6.826	<0.001
Performance→ Competence	0.330	0.064	4.260	<0.001

**Fig. 1:**
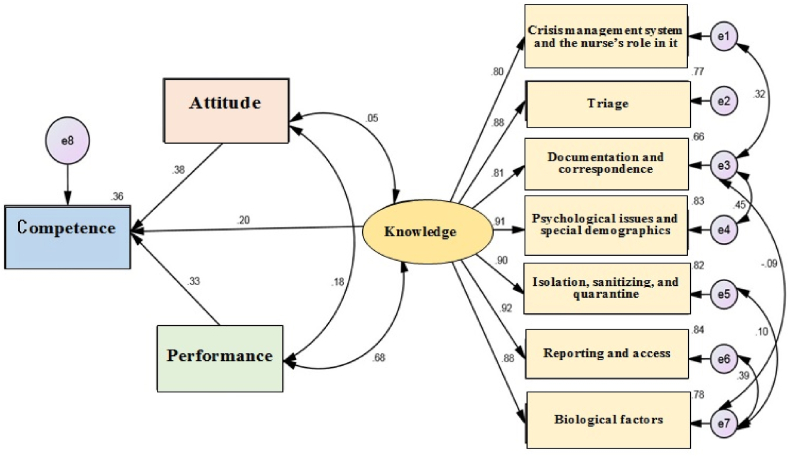
The standard coefficients and the structural relationship between knowledge, attitude and performance with nurses' competence.

Fig. 1 presents the final (modified) models of the research. The final model's direct relationships with research variables indicate that path coefficients are statistically significant, and there is a positive and significant relationship between all research variables in the entire sample.

## 4 Discussion

This study aimed to model the relationship between knowledge, attitude, and performance with the competence of nurses caring for patients with COVID-19 using structural equations. The current study revealed that the mean score of knowledge of more than 50 % of the participants was at an average level. The results of Ghezeljeh et al.’s study (2019) are in line with the present study, stating that nurses play an imperative role in helping people deal with disasters, requiring sufficient knowledge [31]. In another study, it was reported that the mean score of nurses' preparedness to cope with disasters was at a low level; consequently, they concluded that due to the significant role that nurses play in disaster preparedness, such as educating people to reduce vulnerability to disasters and work in a disaster, it is necessary for them to enjoy sufficient preparedness for disaster management upon the occurrence of the specific event [32]. The results of a systematic review indicate that in some developing countries, nurses have low to moderate preparedness concerning disaster management. In contrast, nurses in developed countries have an optimal level of preparedness and knowledge [13]. Hence, Iran's healthcare policymakers and executive managers should create a strategic plan to improve hospitals' preparedness for incidents through training and exercises to enhance risk perception [33].

Additionally, the present study results demonstrated the nurses' attitudes about preparedness for responding to disasters were appropriate, which is in line with several studies. It is believed that the education provided by the Hospital Incident Management team has improved nurses' attitudes, and this positive attitude is probably due to Iran's proneness to disaster [31]. The mean score of nurses' attitudes in Ibrahim's study in Saudi Arabia was at an acceptable level [12]. Those results are in contrast to the results of Soltani et al.’s study (2016), and increasing disaster management training courses for nurses as the largest providers of information and health services to people in times of disaster has been emphasized [9].

Furthermore, the study found that the nurses were well-prepared to respond to disasters, with a high mean score for their performance. In a study, the mean score of participants' performance was below moderate and at a low level. According to the learning/teaching preferences, theoretical and practical training courses related to disasters and preparedness for disaster were essential in nursing curricula [12]. In another study, the mean score of participants’ performance was reported to be weak, and participation in training courses was highlighted [34]. Hence, the Organization for Economic Co-operation and Development specifically notes that when designing nursing curricula, it is of particular importance to ensure that future generations of nurses will have the appropriate competencies to be “fit for practice” [35], and nursing graduates will need education and training conforming to critical changes in the field of health, such as epidemics [28].

Another result indicated that the mean score of competence in more than two-thirds of the participants was at an acceptable level. Labrague et al.’s study (2018) indicated that nurses' competence in responding to crises was below the optimal level [13]. In another study, the mean score of nurses' perceived competence was below the moderate level, and it was stated that most nurses were not confident in their abilities to respond to major crises [23]. Therefore, acquiring competence to provide care in critical conditions is one of the crucial issues in the nursing profession, and technical, managerial, ethical, and personal competencies are essential for all nurses to effectively provide care when a disaster occurs [36]. Consequently, it is required to include training in nurses' university curricula and in-service courses. One of the reasons for the participants' acceptable mean scores of knowledge, attitude, and performance in the present study for disaster management during the COVID-19 pandemic seems to be the use of successful disaster management models since disaster management is considered a long-term, complex, and technical process, the success of which requires scientific knowledge, attitude, and performance. Some studies in this field have shown that transforming hospitals into COVID-19 centers has improved the managerial performance and control of the situation, helped to organize the conditions, and improved health team members' preparedness [37].

The other result was that 88 % of nurses were responsible for taking care of patients in disaster, and only 10 % were not exposed to critical situations. The results of a study indicate that the positive correlation between previous experience and higher scores in emergency preparedness confirms that receiving education or participating in actual events increases nurses' perceived and actual preparedness abilities. Therefore, creating opportunities for nurses to participate in disaster planning and actual exercises and events will increase their competence in disasters, confidence in abilities, and familiarity with disaster preparedness, which can also be a beneficial investment in case of a disaster at a local level [23]. Moreover, while more than 80 % of the participants of this study participated in disaster management exercises, only about 28 % received an official disaster management certificate. More than 80 % felt the need to receive training, and more than half received face-to-face training. Over 60 % considered the need for maneuvers and exercises, and more than 70 % considered nursing schools and the hospital's education department responsible for training. Although more than 50 % of the participants participated in a course related to disaster management during their studies, they felt they had received inadequate training in this field. Some researchers believe that disaster-related and continuous training is an imperative strategy for nurses' better preparedness against a disaster, which improves their capacity in all stages of the disaster, including preparedness, response, recovery, and assessment. To this end, training sufficient nursing staff in this field is required [32]. In addition, the role of education as an effective method to improve nurses' knowledge and skills, holding maneuvers and exercises in the actual environment, participating in institutions' disaster planning, and providing learning opportunities such as simulation methods and models to prepare nurses to respond to the critical situation must not be neglected [13].

Spearman's correlation matrix showed a positive and significant relationship between knowledge, attitude, and performance with nurses' competencies. Besides, the path analysis results revealed several valuable findings regarding the influence of these variables on nurses' competence. Firstly, the analysis showed a strong positive relationship between knowledge and competence (β = 0.205, p = 0.012). Hence, nurses who have a higher level of knowledge are likely to show better competence in their practice. To be precise, for every one-unit increase in the knowledge, there is a corresponding increase of 0.205 units in the competence. This finding highlights the significance of education and training programs that can help improve nurses' knowledge base, as it directly impacts their overall competence in providing quality care to their patients [38]. Secondly, positive attitudes in nurses are strongly associated with higher levels of competence (β = 0.380, p < 0.001). A nurse's attitude plays a crucial role in shaping their performance and ability to provide effective patient care in a disaster [39]. Therefore, fostering a positive work environment and culture of professionalism can significantly enhance overall competence levels. Finally, high levels of performance in nursing duties indicate greater competence (β = 0.330, p < 0.001). Every one-unit increase in performance corresponds to a 0.330-unit increase in competence, which highlights the importance of continuous performance evaluation and feedback mechanisms for professional development support [40]. Considering the path coefficients, the final model showed that knowledge, attitude, and performance could predict and positively affect the changes in nurses' competencies. Almost no study has addressed a similar model; however, in some studies, the significant relationship between knowledge, attitude, and performance and their determining role in nurses' competencies has been pointed out [41,42]. A study also stated that to obtain an acceptable level of competence, an adequate level of proficiency in knowledge, skills, and attitude must be achieved through practical courses [43]. These results can guide managerial decisions, optimize resource allocation, and help design nurse training programs.

Finally, the results related to the relationship between the demographic variables and the study's main variables indicated that the mean score of knowledge, attitude, and performance of nurses above 37 years of age was better than that of younger-age nurses, which is consistent with the results of the study by Rastogi et al. This result seems rational since experience and knowledge develop with age [13,42]. However, it is inconsistent with some studies [[44], [45], [46]], probably because younger nurses have acquired more knowledge due to more awareness and broader access to the media [47]. In contrast to the mean score of attitude, which was higher in women, men outscored women in knowledge, which was consistent with the results of several studies [9,42,46] and inconsistent with others [44,45]. The mean score of nurses' performance with a postgraduate degree was better than that of undergraduate students. A similar result was found in other studies [42,45]. Since the number of nurses with a bachelor's degree is higher in the ward for patients with COVID-19, this can be a warning for managers and requires more focus on training these nurses. The mean score of supervisors' knowledge, attitude, and performance was higher than that of other nurses. No study with similar results was found. Since supervisors are selected from among nurses with work experience or higher education, these results seem rational. The nurses' knowledge, attitude, performance, and competence levels were higher as their work experience increased. These results were consistent with the results of previous studies [46]. In conclusion, there was a variety of correlations between the demographic variables and the main variables of the study that could be influenced by various context-related factors, requiring nurse managers to address these differences locally and nationally and focus on them intensely.

It is important to note that this study was designed as a cross-sectional research that generated correlational data. As a result, it is not possible to draw cause-and-effect conclusions from the data. Furthermore, no uniform questionnaire was utilized to assess the knowledge, attitude, performance, and competence of nurses caring for patients with COVID-19 regarding disaster management. As a result, a standardized tool should be created and validated in future research to establish its dependability.

## 5 Conclusion

This study aimed to model the relationship between knowledge, attitude, and performance with the competence of nurses caring for patients with COVID-19 using structural equations. The results of this study indicated that the mean scores of knowledge, attitude, performance, and competence of these nurses were at an average and acceptable level. Nevertheless, there were obstacles to improving their competence, which required the comprehensive support of nursing policy-makers and encouraging nurses to participate in the actual maneuvers and exercises. Moreover, the positive and significant correlation between knowledge, attitude, and performance and their acceptable influence on the nurses' competence shows that preparedness for managing emergencies is a necessity for nurses as frontline responders in crises and disasters. By identifying and prioritizing nurses' educational needs and learning preferences in this field, effective measures can be taken through a regular and planned approach to train and strengthen nurses' ability to respond appropriately. The results related to the relationships between the main study variables and the demographics are at the national level. Cultural and contextual differences can put nurses in different countries to various challenges, which require further studies. Besides, nurses should always be highly aware of their roles during disasters, be prepared for critical situations, and apply their management skills to handle different clients and situations. Therefore, health organization managers must provide conditions for high professionalism in nurses, as this is their principal role. Moreover, disaster preparedness is a long-term development program that requires more attention to train efficient nurses for crisis management. To address limitations in structural equation modeling, larger sample sizes are suggested in future studies. Additionally, invariance tests can improve the model's difference in demographic variables.

## Funding statement

None.

## Ethics considerations

The study was performed under the Declaration of Helsinki. The code of ethics (IR.KMU.REC.1400.173) was obtained from the Research Ethics Committee of Kerman University of Medical Sciences. All participants completed the electronic, written consent form and were assured that their information would be confidential and anonymous and analyzed only for research purposes. All methods were carried out following relevant guidelines and regulations. All participants were fully informed about the study's purpose. Also, the authors attested that there would be no harm in participating or not participating in the study. Written consent was obtained from all subjects.

## Data availability statement

Data will be made available on request.

## CRediT authorship contribution statement

**Maryam Khandan:** Writing – review & editing, Writing – original draft, Visualization, Validation, Supervision, Software, Resources, Project administration, Methodology, Investigation, Formal analysis, Data curation, Conceptualization. **Zinab Ghorbani:** Writing – review & editing, Validation, Resources, Project administration, Methodology, Investigation, Data curation, Conceptualization. **Mohsen Golestani:** Writing – review & editing, Validation, Supervision, Resources, Methodology, Investigation, Data curation, Conceptualization. **Faranak Moradi:** Writing – review & editing, Validation, Project administration, Methodology, Investigation, Data curation, Conceptualization.

## Declaration of competing interest

The authors declare that they have no known competing financial interests or personal relationships that could have appeared to influence the work reported in this paper.
